# Comparative epidemiology of bacteraemia in two ageing populations: Singapore and Denmark

**DOI:** 10.1017/S0950268824000645

**Published:** 2024-04-29

**Authors:** Patrick Jian Wei Sim, Zongbin Li, Aung Hein Aung, John Eugenio Coia, Ming Chen, Stig Lønberg Nielsen, Thøger Gorm Jensen, Jens Kjølseth Møller, Ram Benny Dessau, Pedro Póvoa, Kim Oren Gradel, Angela Chow, for the SG-DK Bacteraemia study group

**Affiliations:** 1 Department of Preventive and Population Medicine, Office of Clinical Epidemiology, Analytics, and Knowledge (OCEAN), Tan Tock Seng Hospital, Singapore; 2 Department of Clinical Microbiology, Esbjerg Hospital, Esbjerg, Denmark; 3 Department of Regional Health Research, University of Southern Denmark, Odense, Denmark; 4 Department of Clinical Microbiology, Hospital of Southern Jutland, Sønderborg, Denmark; 5 Department of Infectious Diseases, Odense University Hospital, Odense, Denmark; 6 Department of Clinical Research, University of Southern Denmark, Odense C, Denmark; 7 Department of Clinical Microbiology, Odense University Hospital, Odense C, Denmark; 8 Department of Clinical Microbiology, Vejle Hospital, University Hospital of Southern Denmark, Vejle, Denmark; 9 Department of Clinical Microbiology, Zealand University Hospital, Slagelse, Denmark; 10 The Polyvalent Intensive Care Unit, Hospital de São Francisco Xavier, CHLO, Estrada do Forte do Alto do Duque, Lisbon, Portugal; 11 NOVA Medical School, New University of Lisbon, Lisbon, Portugal; 12 Center for Clinical Epidemiology, Odense University Hospital, Odense, Denmark; 13 Infectious Diseases Research and Training Office, National Centre for Infectious Diseases, Singapore; 14 Lee Kong Chian School of Medicine, Nanyang Technological University, Singapore; 15 Saw Swee Hock School of Public Health, National University of Singapore, Singapore

**Keywords:** bloodstream infections, gram-negative bacteria, gram-positive bacteria, hospital microbiology, public health microbiology

## Abstract

Burden of bacteraemia is rising due to increased average life expectancy in developed countries. This study aimed to compare the epidemiology and outcomes of bacteraemia in two similarly ageing populations with different ethnicities in Singapore and Denmark. Historical cohorts from the second largest acute-care hospital in Singapore and in the hospitals of two Danish regions included patients aged 15 and above who were admitted from 1 January 2006 to 31 December 2016 with at least 1 day of hospital stay and a pathogenic organism identified. Among 13 144 and 39 073 bacteraemia patients from Singapore and Denmark, similar 30-day mortality rates (16.5%; 20.3%), length of hospital stay (median 14 (IQR: 9–28) days; 11 (6–21)), and admission rate to ICU (15.5%; 15.6%) were observed, respectively. *Escherichia coli*, *Klebsiella pneumoniae*, and *Staphylococcus aureus* ranked among the top four in both countries. However, Singaporeans had a higher proportion of patients with diabetes (46.8%) and renal disease (29.5%) than the Danes (28.0% and 13.7%, respectively), whilst the Danes had a higher proportion of patients with chronic pulmonary disease (18.0%) and malignancy (35.3%) than Singaporeans (9.7% and 16.2%, respectively). Our study showed that top four causative organisms and clinical outcomes were similar between the two cohorts despite pre-existing comorbidities differed.

## Introduction

Singapore is a tropical city-state located near the equator in Southeast Asia, with a population of 4.4 million in 2006 [1] and 5.6 million in 2016 [1] and 2022 [1], living in a compact area of 733 km^2^. The resident population is multi-ethnic with three major ethnic groups, of which 74.3% were Chinese, 13.4% Malays, and 9.1% Indians in 2016 [1].

In contrast, Denmark is a temperate country located in Northern Europe with a population of 5.4 million in 2006 [2], 5.7 million in 2016 [2], and 5.8 million in 2022 [2], living over an area of 42 493 km^2^. The majority, 85.6%, are ethnically Danish [2].

Bacteraemia occurs as commonly as stroke with an estimated incidence of between 113 and 220 per 100 000 person-years [3] in North America and Europe. Bacteraemia is associated with an increased length of stay, higher rates of readmission, poor clinical outcomes and mortality, and a high economic burden [3, 4]. Elderly patients with co-morbidities, particularly malignancies, are at a higher risk of bacteraemia [3, 5]. The incidence of bacteraemia is expected to increase, with ageing populations and increased longevity of patients with chronic conditions due to medical advancements [3, 5]. Both Singapore and Denmark have rapidly ageing populations. In Singapore, 12.4% and 18.4% of the population were aged 65 years and above in 2016 [1] and 2022 [1], respectively, and estimated to increase to 23.8% by 2030 [1]. Similarly, Denmark had a high proportion of elderly with 19% and 20.3% of the population aged 65 years and above in 2016 [2] and 2021 [2], respectively. Whilst the epidemiology of bacteraemia in various developed countries has been described, more studies on the comparative epidemiology and outcomes of bacteraemia between countries are needed [3] to understand the dynamics behind geographical variation in the epidemiology of bacteraemia.

In this study, we compared and contrasted the epidemiology and outcomes of bacteraemia in similarly sized ageing populations in two developed countries across two continents: Singapore, a multi-ethnic Asian country, and Denmark, a relatively ethnically homogenous European country.

## Methods

### Study setting

The Singapore cohort (SG) was conducted in Tan Tock Seng Hospital, a publicly funded 1 600-bed tertiary-care hospital located in the central region of Singapore. It is the second largest hospital serving about 1.4 million residents, representing 25% of Singapore’s population, with healthcare services heavily subsidized by the government. The electronic health records and administrative databases at the hospital were accessed for the study and linked with the use of a unique personal identifier. In Denmark, the residents have access to free healthcare with the Danish national health system being fully funded by taxpayers, covering both primary and hospital care [6]. The Denmark cohort (DK) was conducted using data from the SydBak research database, a population-based health database comprising all bacteraemia episodes captured in two of Denmark’s five regions, Region Zealand, and the Region of Southern Denmark [6]. A unique personal identifier served as a linkage between various healthcare administrative registries [6] in Denmark. The two regions comprise 2 023 000 inhabitants, representing 35% of Denmark’s population [2]. This well-defined catchment area for acute admissions at all hospitals in the two regions enables population-based studies.

### Patient cohort, inclusion, and exclusion criteria

Patients aged 15 and above hospitalized with at least one bacteraemia episode between 1 January 2006 and 31 December 2016 were included. All blood cultures with bacterial or fungal growths were included for the analysis. Contaminants (coagulase-negative staphylococci, *Bacillus* spp., *Propionibacterium* spp., *Corynebacterium* spp., viridans group streptococci, *Aerococcus* spp., or *Micrococcus* spp.) were not included unless they were isolated in ≥2 blood cultures within 5 days [7]. Where a patient had multiple admissions, only the index (first) admission was included with the date of the first positive blood culture defined as the date of bacteraemia episode. We defined poly-microbial bacteraemia as two or more different micro-organisms isolated not more than 2 days apart during an admission [8]. Four patients were excluded for analysis of 30-day mortality due to missing death dates.

### Outcome variables and covariates

30-day mortality (defined as death occurring within 30 days from the date of bacteraemia episode), length of hospital (LOS) stay, and admission to an intensive care unit were outcomes of the bacteraemia episode.

The covariates collected included demographic characteristics (age and gender), presence of co-morbidity, type of acquisition, and the number and type of organisms grown in blood cultures.

The comorbidities were determined based on discharge diagnosis codes and computed the Charlson Comorbidity Index as defined by Quan et al. [9]

Type of acquisition was defined using globally accepted definitions, considering data limitations. A nosocomial infection was defined as a bacteraemia episode with the first positive blood culture occurring more than 48 h after hospital admission [10]. Or, as a likely healthcare-associated infection (HCA) if the first positive blood culture was within 48 h of hospital admission for patients with previous hospitalization in the past 30 days [10]. All remaining bacteraemia were classified as community acquired.

### Statistical analysis

The cohorts were described based on the patient demographics, comorbidities, type of acquisition, number and type of organisms grown in blood cultures, and clinical outcomes. Descriptive statistics were performed in both cohorts using STATA to obtain frequency, mean, and range for normally distributed variables or median and interquartile range (IQR) for non-normally distributed variables. Data of interest and importance are bolded under results and further examined in discussion.

### Ethical considerations

In Singapore, ethics approval was obtained prior to the study, with waiver of consent given as the study involved retrospective review of medical records without any patient contact (Reference number: 2017/00246). In Denmark, as the studies were registry-based and without patient contact, approval from an ethics committee or consent from participants is not required. However, because microbiology data are legally considered to be medical chart data, we needed permission for using these (Danish Patient Safety Authority, rec. no. 3-3013-945/1 & 3-3013-945/2). A Data Processor Agreement was completed between the two countries (rec. no. 18/57806).

## Results

A total of 51 359 patients, 13 144 from Singapore and 39 073 from Denmark, were included in the study (Table 1).

**Table 1.** Patient demographics, outcomes, and details of bacteraemia admission

	Singapore	Denmark
*Demographics*		
No. of patients	13 144	39 073
Age (median, IQR)	71 (60–81)	72 (62–81)
Gender (*n*, %)		
Female	6 122 (46.6)	16 970 (43.4)
Male	7 022 (53.4)	22 103 (56.6)
Charlson Comorbidity Index (median, IQR)	3 (1–5)	2 (1–4)
0 (*n*, %)	2 123 (16.2)	7 711 (19.7)
1–2	3 942 (30.0)	14 378 (36.8)
>2	7 079 (53.9)	16 984 (43.5)
Comorbidities (*n*, %)		
Diabetes mellitus (any)	6 155 **(46.8)**	10 958 (28.0)
Without complication	1 693 (12.9)	6 954 (17.8)
With complication	4 462 (33.9)	4 004 (10.3)
Renal disease	3 883 **(29.5)**	5 337 (13.7)
Cerebrovascular disease	2 856 (21.7)	7 441 (19.0)
Myocardial infarction	2 261 (17.2)	4 505 (11.5)
Malignancy (any)	2 133 (16.2)	13 802 **(35.3)**
Localized	1 106 (8.4)	11 314 (29.0)
Metastatic solid tumour	1 027 (7.8)	2 488 (6.4)
Congestive heart failure	1 871 (14.2)	5 282 (13.5)
Liver disease	1 435 (10.9)	3 344 (8.6)
Mild	1 074 (8.2)	2 132 (5.5)
Moderate/severe	361 (2.8)	1 212 (3.1)
Chronic pulmonary disease	1 276 (9.7)	7 023 **(18.0)**
Dementia	1 098 (8.4)	1 714 (4.4)
Peripheral vascular disease	1 107 (8.4)	3 941 (10.1)
Peptic ulcer disease	876 (6.7)	3 902 (10.0)
Hemi/paraplegia	639 (4.9)	447 (1.1)
Connective tissue disease	276 (2.1)	2 401 (6.1)
Acquired immunodeficiency syndrome	158 (1.2)	73 (0.2)
Lymphoma	151 (1.2)	1 526 (3.9)
Leukaemia	132 (1.0)	1 034 (2.7)
*Outcomes*		
30–day mortality (*n*, %)	2 169 (16.5)	7 938 (20.3)
Overall length of stay, days (median, IQR)	14 (9–28)	11 (6–21)
Required intensive care unit admission (*n*, %)	2 041 (15.5)	6 078 (15.6)
*Type of bacteraemia*		
Acquisition (*n*, %)		
Community–acquired	8 709 (66.2)	19 773 (50.6)
Nosocomial infections	3 158 (24.0)	7 009 (17.9)
Healthcare–associated	1 277 (9.7)	11 994 (30.7)
Unknown	0 (0.0)	297 (0.8)
No. of organisms within episode (*n*, %)		
Mono–microbial	11 337 (86.3)	35 283 (90.3)
Poly–microbial	1 807 (13.8)	3 790 (9.7)

Numbers highlighted in bold are marked as data of interest for further discussion. IQR, interquartile range.

The yearly numbers of admission were around 57 000 between 2006 and 2016 with the yearly incidence estimated to be hovering around 2.4% in Singapore. Compared to Denmark, the yearly numbers of admissions were around 420 000 between 2006 and 2016 with the yearly incidence estimated to be around 1.8%, which was comparable to Singapore. Patients from Singapore and Denmark had similar distributions of age, gender, and Charlson Comorbidity Index. However, Singapore had a higher prevalence of diabetes, renal disease, and myocardial infarction, while Denmark had a higher prevalence of malignancies and chronic pulmonary disease.

The 30-day mortality of the first positive blood culture was 16.5% in Singapore and 20.3% in Denmark. The length of hospital stay (Denmark, median 11 days, interquartile range (IQR): 6–21; Singapore, median 14 days, IQR: 9–28) and admissions to ICU (Denmark, 15.6%; Singapore, 15.5%) among bacteraemia patients were also similar between the two countries.

The Singaporean cohort comprised of more community-acquired bacteraemia admissions (SG: 66.2% vs. DK: 50.6%), whereas the Danish cohort had more healthcare-associated bacteraemia admissions (SG: 9.7% vs. DK: 30.7%), whereas nosocomial episodes were similar.

*Escherichia coli, Klebsiella pneumoniae*, and *Staphylococcus aureus* were similarly common causative organisms of bacteraemia in Singapore and Denmark, ranking among the top four in both countries (Table 2). Whilst *K. pneumoniae* was the second most common cause of bacteraemia in Singapore, *Streptococcus pneumoniae* was more common in Denmark. The top ten organisms represent the 78.4% of the Singapore and 73.1% of the Denmark cohort.

**Table 2.** Top ten organisms from admissions with mono-microbial bacteraemia (species) from the Singapore and Danish cohort (*n*, %)

Rank	Singapore	*n* (%)	Denmark	*n* (%)
1	*Escherichia coli*	3 996 (35.3)	*E. coli*	11 154 (32.3)
2	*Klebsiella pneumoniae*	1 795 (15.8)	*Staphylococcus aureus*	4 220 (12.2)
3	*S. aureus*	1 556 (13.7)	*Streptococcus pneumoniae*	2 580 (7.5)
4	*Pseudomonas aeruginosa*	319 (2.8)	*K. pneumoniae*	1 927 (5.6)
5	*Salmonella Enteritidis*	236 (2.1)	*Staphylococcus epidermidis*	1 239 (3.6)
6	*Streptococcus agalactiae (Group B streptococcus)*	229 (2.0)	*Enterococcus faecalis*	1 059 (3.1)
7	*Proteus mirabilis*	227 (2.0)	*Enterococcus faecium*	899 (2.6)
8	*S. pneumoniae*	201 (1.8)	*P. aeruginosa*	887 (2.6)
9	*Acinetobacter baumannii*	180 (1.6)	*Coagulase–negative staphylococci*	729 (1.8)
10	*Enterobacter cloacae*	144 (1.3)	Haemolytic streptococci, Group G	626 (1.8)

## Discussion

This study contemporaneously compared the epidemiology and clinical outcomes of bacteraemia in a temperate and tropical country in Europe and Asia, of similar population sizes. Despite differences in the climate, geography, and ethnicities, the top causative organisms were similar, with *E. coli*, *S. aureus*, and *K. pneumoniae* being among the top four.

Interestingly, *Salmonella Enteritidis* and *Streptococcus agalactiae* were among the top ten pathogens in Singapore but not in Denmark. *Salmonella* infections were more common in Singapore where the incidence rate of non-typhoidal salmonellosis in 2016 was 39.4 per 100 000 population [11] as compared to 18.8 per 100 000 in Denmark [12]. Warmer tropical waters (27°C) were associated with increased pathogenicity of *S. agalactiae* [13] infections in fish with lower mortality but was not observed in colder waters (22°C) due to lower expression of virulence genes [13]. Consumption of virulent strains such as the sequence type 283 (ST283) which was prevalent in farm fishes across Southeast Asian region caused healthy adults to be susceptible to ST283 [14] infection.

Although the age–gender distributions were very similar between the bacteraemia cohorts compared, the underlying comorbidities were dissimilar. The Singaporean cohort had a higher proportion of patients with diabetes (46.8%) and renal disease (29.5%) than the Danish cohort (28.0% and 13.7%, respectively). This is reflective of the differences in the chronic disease epidemiology in the two populations. In Singapore, the prevalence of diabetes among adults over 18 years of age has doubled from 4.7% in 1980 to 8.5% in 2014, prompting the Ministry of Health to declare a ‘War on Diabetes’ in 2016 [15]. The prevalence of diabetes in Singapore was 8.8% in 2017 [16] with an age-standardized prevalence rate of chronic kidney disease (CKD) of 9 333 per 100 000 [17]. In contrast, the prevalence of diabetes in Denmark was 6.5% in 2017 [18] with an age-standardized CKD prevalence rate of 5 816 per 100 000 [17]. The differences in the prevalence of diabetes and renal disease between the two populations could be due to differences in lifestyles such as diet and physical activity in the two countries.

The Danish cohort (35.4%) had more than double the prevalence of malignancy than the Singaporean cohort (16.2%). Denmark has been reported by the World Cancer Research Fund to have one of the highest cancer rates in the world, with 334.9 persons per 100 000 population in 2020 [19]. In comparison, Singapore’s age-standardized cancer incidence rate was reported to be 235.0 per 100 000 population in 2015–2019 [20]. Cancer patients have been shown to have a higher risk of acquiring bloodstream infections, especially patients with haematological malignancies [4]. Whilst diabetes and renal disease may be predisposing Singaporeans to bacteraemia, malignancy may be the predisposing factor for Danes.

Notably, the Danish cohort had a higher proportion of patients with COPD (18.0%) than the Singaporean cohort (9.7%). This reflects the higher smoking prevalence in Denmark (21.1% in 2015) than in Singapore (16.5% in 2015) [21]. It is well established that smoking is associated with the development of COPD, which is in turn associated with the increased risk of lung infection [22]. This is corroborated by the finding that *S. pneumoniae*, a respiratory pathogen, is more common in the Danish cohort (7.4%) than the Singaporean cohort (1.8%). In comparison, *K. pneumoniae* was more common in the Singaporean cohort than the Danish cohort. *K. pneumoniae* has been previously observed to be associated with pneumonia in Asian patient populations with diabetes mellitus [23], consistent with our observation of a higher prevalence of diabetes mellitus in the Singaporean bacteraemia cohort.

The clinical outcomes, in particular, 30-day mortality (16.5% and 20.3%) in both cohorts, were similar despite differences in distribution of pre-existing comorbidities between them. Other developed countries have reported similar mortality rates of 13%–19%, although developing countries have reported rates of up to 37.5% [24, 25]. This clearly reflects the advanced healthcare systems in both countries [26], despite similarly older patient cohorts who were at increased risk of mortality from bacteraemia. Further research is needed to explore the predictors of mortality from bacteraemia in Singapore and Denmark.

### Future implications for research

The data presented in this study only reflect some of the data in the two cohorts. Data on biomarkers of bacteraemia are available for all Singaporean patients and for the Danish patients hospitalized in the Region of Southern Denmark. The unique patient identifier number enabled linkages to other registries, which has been previously done for patients with haematological malignancies [4]. Ongoing studies with the two cohorts include readmissions after bacteraemia, nomograms that prioritize predictors of mortality, and derivation of biomarker clusters by machine learning methods inspired by a study on sepsis [27]. In the near future, we plan to embark on studies of longitudinal measurements of blood biomarkers in relation to inflammation, such as C-reactive protein or albumin and prospective study in understanding how other risk factors such as the use of catheter lines and antibiotic resistance patterns are different in patients with bacteraemia in both countries.

### Strengths

Both Singapore and Denmark have healthcare systems with unique electronic medical records, allowing for the accrual of the respective 10-year bacteraemia cohorts and the longitudinal follow-up for clinical outcomes. Both healthcare systems were able to identify high numbers of bacteraemia episodes via positive blood culture and retrieve relevant patients’ records with good accuracy. Such high coverage settings in the study sites in both countries (25% and 35%) allowed population-based studies to be established which can be used to determine the incidence rate of bacteraemia and the burden of bacteraemia in the population over time, monitoring the proportion of community-acquired, nosocomial, and healthcare-associated bacteraemia, and detecting a shift in the frequency of microbial isolates [28].

Bacteraemia is a serious infection of bloodstream, a sterile site. Bacteraemia is different from sepsis, which can be complicated by different classification of diseases code abstraction strategies and interpretation by different professions [29]. This may lead to misclassification bias which in turn underestimates the true incidence of sepsis. However, bacteraemia is a microbiological finding which is clear and precise. The same definitions for bacteraemia and laboratory classification in categorizing microorganisms were used for both cohorts, minimizing any misclassification bias. Furthermore, all first admission episodes for bacteraemia were included in the study and followed up prospectively for 30 days after the positive blood culture, rendering any selection bias negligible.

### Limitations

The main limitation is the lack of clinical data, which is a general weakness of data based on administrative registries though co-morbidities were coded from clinical data into diagnosis indexes, thus should not affect the accuracy on detection of co-morbidities conditions. Further, merging of the two cohorts’ data would be advantageous, but this is not possible due to legislation, but future studies will use one cohort for derivation and another for validation. Also, HCA’s definition includes residence in nursing and elderly folks’ home or regular home care by a nurse which were absent in both cohorts’ data.

The study could be limited by the non-generalizability of findings to the Singaporean population, as only one hospital was included. However, the patient demographic of the study site is known to be 10 years older than those of other public hospitals in Singapore. As such, they present the profile of the population with the highest risk for poor clinical outcomes. Thus, understanding the epidemiology of bacteraemia in this vulnerable elderly group would enable the better management of bacteraemia in an ageing population.

## Conclusion

The study presents important insights into the epidemiology and clinical outcomes of bacteraemia contemporaneously in two similarly sized rapidly ageing populations in two developed countries across two continents. Whilst the top four causative organisms and clinical outcomes were similar, the pre-existing comorbidities differed between the two cohorts. More efforts in diabetes prevention and smoking cessation could be promoted in Singapore and Denmark, respectively, to reduce the burden of bacteraemia.

## Data Availability

The final dataset is partially available on request.

## Authors’ information

Contributing members of the SG-DK Bacteraemia study group include Patrick Sim Jian Wei, Li Zongbin, Aung Aung Hein, John Eugenio Coia, Ming Chen, Stig Lønberg Nielsen, Thøger Gorm Jensen, Jens Kjølseth Møller, Ram Benny Dessau, Pedro Póvoa, Kim Oren Gradel, Angela Chow, Olav Sivertsen Garvik, and Caroline Thingholm Thorarinsson.

## References

[1] Department of Statistics, Singapore (2023) SingStat Table Builder. Available at https://tablebuilder.singstat.gov.sg/table/TS/M015651#! (accessed 14 March 2023).

[2] Statistics Denmark (2023) Population Figures. Available at https://www.dst.dk/en/Statistik/emner/borgere/befolkning/befolkningstal (accessed 15 March 2023).

[3] Kern WV, Rieg S (2020) Burden of bacterial bloodstream infection—a brief update on epidemiology and significance of multidrug-resistant pathogens. Clinical Microbiology Infection 26, 151–157. 10.1016/j.cmi.2019.10.031 31712069

[4] Gradel KO, et al. (2020) Longitudinal trajectory patterns of plasma albumin and C-reactive protein levels around diagnosis, relapse, bacteraemia, and death of acute myeloid leukaemia patients. BioMed Central Cancer 20, 249. 10.1186/s12885-020-06754-z 32209087 PMC7092519

[5] Payeras A, et al. (2007) Bacteriemia en pacientes muy ancianos: factores de riesgo, características clínicas y mortalidad (Bacteraemia in very elderly patients: Risk factors, clinical characteristics and mortality) (in Spanish). Enfermedades Infecciosas y Microbiología Clínica 25, 612–618. 10.1157/13112936 18053471

[6] Schmidt M, et al. (2019) The Danish health care system and epidemiological research: From health care contacts to database records. Clinical Epidemiology 11, 563–591. 10.2147/CLEP.S179083. PMID: 31372058; PMCID: PMC6634267. 31372058 PMC6634267

[7] Horan TC, et al. (2008) CDC/NHSN surveillance definition of health care-associated infection and criteria for specific types of infections in the acute care setting. American Journal of Infection Control 36,309–332. 10.1016/j.ajic.2008.03.002. Erratum in (2008) *American Journal of Infection Control* **36**(9), 655. 18538699

[8] Roberts FJ (1989) Definition of polymicrobial bacteraemia. Review of Infectious Disease 11, 1029–1030. 10.1093/clinids/11.6.1029 2602769

[9] Quan H, et al. (2005) Coding algorithms for defining comorbidities in ICD-9-CM and ICD-10 administrative data. Medical Care 43, 1130–1139. 10.1097/01.mlr.0000182534.19832.83 16224307

[10] Friedman ND, et al. (2002) Health care-associated bloodstream infections in adults: A reason to change the accepted definition of community-acquired infections. Annals of Internal Medicine 137, 791–797. 10.7326/0003-4819-137-10-200211190-00007 12435215

[11] Ministry of Health Singapore (2023) Communicable Diseases Surveillance in Singapore 2016. Available at https://www.moh.gov.sg/docs/librariesprovider5/resources-statistics/reports/full-version.pdf (accessed 19 June 2023).

[12] Statens Serum Institut Denmark (2023) Salmonella Infections, 2016–2017. Available at https://en.ssi.dk/surveillance-and-preparedness/surveillance-in-denmark/annual-reports-on-disease-incidence/salmonella-infections-2016-2017 (accessed 19 June 2023).

[13] Tavares GC, et al. (2018) Transcriptome and proteome of fish-pathogenic Streptococcus agalactiae are modulated by temperature. Frontiers in Microbiology 9, 2639. 10.3389/fmicb.2018.02639 30450092 PMC6224512

[14] Aiewsakun P, et al. (2022) Genomic epidemiology of Streptococcus agalactiae ST283 in Southeast Asia. Scientific Reports 12, 4185. 10.1038/s41598-022-08097-0. 35264716 PMC8907273

[15] Ministry of Health, Singapore (2023) Result from Government’s Five-Year ‘War against Diabetes’ Effort. Available at https://www.moh.gov.sg/news-highlights/details/result-from-government%27s-five-year-war-against-diabetes-effort/ (accessed 20 March 2023).

[16] Ministry of Health, Singapore (2023) National Population Health Survey 16/17. Available at https://www.moh.gov.sg/resources-statistics/reports/national-population-health-survey-2016-17 (accessed 17 March 2023).

[17] GBD Chronic Kidney Disease Collaboration (2020) Global, regional, and national burden of chronic kidney disease, 1990–2017: A systematic analysis for the Global Burden of Disease Study 2017. The Lancet 395, 709–733. 10.1016/S0140-6736(20)30045-3 PMC7049905 32061315

[18] Organisation for Economic Cooperation and Development (OECD) (2018) Diabetes prevalence. In Health at a Glance: Europe 2018: State of Health in the EU Cycle. Paris: OECD Publishing. 10.1787/health_glance_eur-2018-18-en

[19] World Cancer Research Fund International (2023) Global Cancer Data by Country. Available at Global cancer data by country | World Cancer Research Fund International (https://wcrf.org) (accessed on 27 March 2023).

[20] National Registry of Diseases Office, Health Promotion Board, Singapore (2023) Singapore Cancer Registry Annual Report 2019. Available at https://www.nrdo.gov.sg/docs/librariesprovider3/default-document-library/scr-2019_annual-report_final.pdf (accessed 25 March 2023).

[21] Age-Standardized Estimates of Current Tobacco Use, Tobacco Smoking and Cigarette Smoking Data by Country (2023). Available at https://www.who.int/data/gho/data/indicators/indicator-details/GHO/gho-tobacco-control-monitor-current-tobaccouse-tobaccosmoking-cigarrettesmoking-agestd-tobagestdcurr (accessed 8 June 2023).

[22] Trosini-Desert V, et al. (2004) Exposition à la fumée du tabac et risque infectieux bactérien (Tobacco smoke and risk of bacterial infection) (in French). La Revue des Maladies Respiratoires 21, 539–547. 10.1016/s0761-8425(04)71358-3 15292846

[23] Huang CH, et al. (2015) Risk factors for in-hospital mortality in patients with type 2 diabetes complicated by community-acquired *Klebsiella pneumoniae* bacteremia. Journal of Formosan Medical Association 114, 916–922. 10.1016/j.jfma.2015.07.011 26315482

[24] Abernethy JK, et al. (2015) Thirty-day all-cause mortality in patients with *Escherichia coli* bacteraemia in England. Clinical Microbiology and Infection 21(3), 251.e1–251.e8. 10.1016/j.cmi.2015.01.001 25698659

[25] Kanoksil M, et al. (2013) Epidemiology, microbiology and mortality associated with community-acquired bacteraemia in Northeast Thailand: A multicenter surveillance study. PLoS One 8, e54714. 10.1371/journal.pone.0054714. Erratum in (2013) *PLoS One* **8**. 10.1371/journal.pone.0054714 23349954 PMC3548794

[26] Axelsson C, et al. (2016) The early chain of care in patients with bacteraemia with the emphasis on the prehospital setting. Prehospital and Disaster Medicine 3, 272–277. 10.1017/S1049023X16000339 27026077

[27] Seymour CW, et al. (2019) Derivation, validation, and potential treatment implications of novel clinical phenotypes for Sepsis. Journal of the American Medical Association 321(20), 2003–2017. 10.1001/jama.2019.5791 31104070 PMC6537818

[28] Laupland KB (2013) Incidence of bloodstream infection: A review of population-based studies. Clinical Microbiology and Infection 19, 492–500. 10.1111/1469-0691.12144 23398633

[29] Wilhelms SB et al. (2010) Assessment of incidence of severe sepsis in Sweden using different ways of abstracting International Classification of Diseases codes: Difficulties with methods and interpretation of results. Critical Care Medicine 38, 1442–1449. 10.1097/CCM.0b013e3181de4406 20400903

